# Expression, purification and characterization of an endoglucanase from *Serratia proteamaculans* CDBB-1961, isolated from the gut of *Dendroctonus adjunctus* (Coleoptera: Scolytinae)

**DOI:** 10.1186/s13568-016-0233-9

**Published:** 2016-08-31

**Authors:** Claudia Cano-Ramírez, Alejandro Santiago-Hernández, Flor Nohemí Rivera-Orduña, Yolanda García-Huante, Gerardo Zúñiga, María Eugenia Hidalgo-Lara

**Affiliations:** 1 Departamento de Biotecnología y Bioingeniería, CINVESTAV-IPN, México City, México; 2 Departamento de Microbiología, Escuela Nacional de Ciencias Biológicas, Instituto Politécnico Nacional, México City, México; 3 Departamento de Zoología, Escuela Nacional de Ciencias Biológicas, Instituto Politécnico Nacional, México City, México

**Keywords:** Cellulase, *Serratia proteamaculans*, Symbiosis, Bark beetles, Gut

## Abstract

*Serratia proteamaculans* CDBB-1961, a gut symbiont from the roundheaded pine beetle *Dendroctonus adjunctus*, displayed strong cellulolytic activity on agar-plates with carboxymethyl cellulose (CMC) as carbon source. Automatic genome annotation of *S*. *proteamaculans* made possible the identification of a single endoglucanase encoding gene, designated *spr cel8A*. The predicted protein, named *Spr* Cel8A shows high similarity (59–94 %) to endo-1,4-β-d-glucanases (EC 3.2.1.4) from the glycoside hydrolase family 8 (GH8). The gene *spr cel8A* has an ORF of 1113 bp, encoding a 371 amino acid residue protein (41.2 kDa) with a signal peptide of 23 amino acid residues. Expression of the gene *spr cel8A* in *Escherichia coli* yields a mature recombinant endoglucanase 39 kDa. Cel8A displayed optimal activity at pH 7.0 and 40 °C, with a specific activity of 0.85 U/mg. The enzyme was stable at pH from 4 to 8.5, retaining nearly 40–80 % of its original activity, and exhibited a half-life of 8 days at 40 °C. The *K*_*m*_ and *V*_*max*_ values for *Spr* Cel8A were 6.87 mg/ml and 3.5 μmol/min/mg of protein, respectively, using CMC as substrate. The final principle products of *Spr* Cel8A-mediated hydrolysis of CMC were cellobiose, cello oligosaccharides and a small amount of glucose, suggesting that *Spr* Cel8A is an endo-β-1,4-glucanase manifesting exo-activity. This is the first report regarding the functional biochemical and molecular characterization of an endoglucanase from *S. proteamaculans*, found in the gut-associated bacteria community of *Dendroctonus* bark beetles. These results contribute to improved understanding of the functional role played by this bacterium as a symbiont of bark beetles.

## Introduction

*Dendroctonus* bark beetles (Curculionidae: Scolytinae) are natural agents of change in forest ecosystems, as they parasitize and kill damaged, stressed or old pine trees (Fam: Pinaceae), thus promoting natural regeneration of the forest. Some species have great ecological and economic importance, as broadscale outbreaks are common, resulting in extensive mortality of healthy trees. Their life cycle largely takes place within tree bark, where larvae and adults consume phloem, a resource rich in both nutrients and complex molecules including cellulose (15–40 %), hemicellulose and pectin (30–40 %), and lignin (20 %) (Doi and Kosugi 2004).

The digestion of these compounds entails a complex process in which numerous enzymes are involved, consisting of a wide spectrum on specific substrate (Payne et al. 2015). In particular, the cellulose is a linear homopolymer of glucose linked by β-1,4 glycosidic bonds, which generates a zigzag arrangement between neighboring oxygen bridges. The enzymes involved in the depolymerization of cellulose, are mainly endoglucanase (EC 3.2.1.4), exoglucanase (EC 3.2.1.176) (EC 3.2.1.91) and β-glucosidase (EC 3.2.1.21). Endoglucanases hydrolyze glycosidic bonds found in the amorphous regions of the cellulose, generating long chain oligomers for the action of exoglucanases that liberate cellobiose. This is the substrate of β-glucosidase, which results in the production of glucose as the end product (Medie et al. 2012).

Whereas it has been shown that some species of insects from the Isoptera, Coleoptera, Orthoptera and Blattodea orders have the ability to digest cellulose (Sugimura et al. 2003; Kim et al. 2008; Willis et al. 2010; Mei et al. 2016), there is no evidence that bark beetles have this capacity. However, it has been demonstrated that different bacteria from the *Cellulomonas*, *Cellulosimicrobium*, *Janibacter*, and *Leifsonia* genera, isolated from the adult guts of *Dendroctonus valens* (Morales-Jiménez et al. 2009), *Stenotrophomonas*, *Ponticoccus*, as well as *Kocuria* isolated from the adult and larval gut of *Dendroctonus rhizophagus* (Morales-Jiménez et al. 2012), and *Serratia*, *Pseudomonas*, *Brevundimona*, *Methylobacterium*, and *Pseudoxanthomonas* isolated from *Dendroctonus armandi* larval gut (Hu et al. 2014) have cellulolytic capacities.

Bacterial cellulases have attracted much attention because of their varied applicability in industrial processes, including food and brewery production, animal food processing, detergent production and laundry, textile processing and paper pulp manufacture (Juturu and Wu 2014). Likewise, because of the crisis concerning the sustainable supply of fossil fuel and the increased demand for production of biofuels and chemicals from renewable resources, their applications in cellulose biorefinery for producing fermentable sugars are expected to rapidly increase in the foreseeable future (Juturu and Wu 2014; Bhat 2000).

*Serratia proteamaculans* CDBB-1961, a gram-negative bacteria, isolated from the adult gut of the roundheaded pine beetle, *D. adjunctus* Blandford, shows outstanding cellulolytic activity in vitro, in comparison to many other bacteria isolated from the guts of other bark beetles (our unpublished results). This activity places *S. proteamaculans* CDBB-1961 as an excellent study model for testing the hydrolytic capacity of its cellulolytic activity. However, whereas many endoglucanases from different *Serratia* spp. have been annotated and deposited in the GenBank (NCBI), to our knowledge there are no studies on the molecular and biochemical characterization of these enzymes. Thus, the present study aims to provide a functional characterization of a novel endoglucanase gene from *S. proteamaculans* in *Escherichia coli*.

## Materials and methods

### Chemicals

Culture media were obtained from BD Difco (Sparks, MD, USA) and JT Baker (Phillipsburg, NJ, USA). All other chemicals, solvents and substrates used were of analytical grade and purchased from Sigma-Aldrich (St. Louis, MO, USA) and JT Baker. Kits for expression, DNA isolation and purification, as well as polymerase enzyme and Ni–NTA resin were obtained from Qiagen (Valencia, CA, USA). Restriction enzymes and DNA ladder were purchased from New England Biolabs (Beverly, MA, USA). pJET1.2/blunt vector was purchased from Fermentas (St Leon-Rot, Germany). The chemicals, including the protein molecular weight markers used in the sodium dodecyl sulfate polyacrylamide gel electrophoresis (SDS-PAGE) and agarose gels analysis and the Econo gradient pump system were purchased from BioRad (Hercules, CA, USA). Lysozyme was obtained from Research Organics (Cleveland, OH, USA) and bovine serum albumin (BSA) was purchased from Pierce (Rockford, IL, USA). Finally, for thin-layer chromatography of silica gel was purchased from Merck KGaA (Darmstadt, Germany).

### Isolation and characterization of the *Serratia proteamaculans* strain

*Serratia* sp. strain DADMA8 was isolated from the gut of *D. adjunctus* emerged-adults, which were removed with chisel and hammer from naturally infested *Pinus hartwegii* trees at 3400 m.a.s.l. in the Sierra del Tigre, Jalisco, México (19^º^35.7′N, 103^º^36′W). From a set of 30-guts, serial ten-fold dilutions of the sample were added to phosphate-buffered-saline (PBS) solution, and 100 μl of each dilution were spread on tryptic soy agar (TSA). Plates were incubated at 28 °C for 48–72 h. Based on their morphological characteristics, 60 colonies of bacteria were randomly isolated from the plates. Axenic cultures were stored at −70 °C in 50 % glycerol. *Serratia* sp. strain DADMA8 was taxonomically identified as *S. proteamaculans*, based on its 16S rRNA sequence (GenBank accession number AJ233434) and whole genome sequence data analysis. *S. proteamaculans* was deposited in the National Collection of Microbial Strains and Cellular Cultures (Colección Nacional de Cepas Microbianas y Cultivos Celulares) from CINVESTAV-IPN, Mexico (accession number CDBB-1961).

### Bacterial strains, plasmids and growth conditions

*Serratia proteamaculans* was grown on Congo red agar plates (0.37 g L^−1^ K_2_HPO_4_, 0.27 g L^−1^ MgSO_4_, 1.88 g L^−1^ carboxymethyl cellulose (CMC), 0.2 g L^−1^ Congo red, 5 g L^−1^ gelatin, 15 g L^−1^ agar) and incubated at 28 °C for 24–48 h.

*Escherichia coli* strains DH5α and the plasmid pJET were used for DNA amplification. *E. coli* strains JM109 and M15 [pREP4], and expression vector pQE30 were used for protein expression, following the manufacturer’s instructions for the QIAexpressionist kit. *E. coli* strains were grown in agar plates and/or Luria–Bertani (LB) medium containing ampicillin (100 μg/mL) and/or kanamycin (30 μg/mL) where appropriate, and then they were incubated at 37 °C overnight, unless otherwise stated.

### Analysis of gene, putatively involved in cellulose degradation

The genome from *S. proteamaculans* CDBB-1961 was sequenced and analyzed by Rapid Annotation Subsystem Technology (RAST) (Aziz et al. 2008) and HMMER 3.1b1 software (http://hmmer.org/). A cellulase encoding gene, named *spr cel8A*, was identified by automatic annotation and deposited in the GenBank (accession number KX023906). The nucleotide sequence of the *spr cel8A* gene was analyzed and compared to sequence databases by using available online tools (http://www.ncbi.nlm.nih.gov/, http://www.expasy.org).

### Analysis of full-length cellulase sequences 

Physicochemical characteristics, including molecular mass and isoelectric point (pI) of the predicted protein were determined using the ProtParam program (Gasteiger et al. 2005). Crystal structure data for the cellulase BcsZ from *E. coli* strain K12 (PDB accession code 3QXF) (http://www.rcsborg/) was used as a template in the ESPript program (Robert and Gouet 2014) to predict the secondary structure elements of *Spr* Cel8A and other cellulases, showing great similarity to this enzyme. The glycosidase hairpin and active sites were manually identified based on information from the sequence template (3QXF). The likely sub-cellular localization of *Spr* Cel8A was determined using the TargetP software (Emanuelsson et al. 2000). The Glycopp V1.0 program was used to predict potential *N*- and *O*-glycosylation sites (Chauhan et al. 2012).

### Cloning of *spr cel8A* cellulose gene

All molecular methods were performed using standard molecular biology techniques (Sambrook et al. 1989). Genomic DNA from *S. proteomaculans* was isolated following the protocol provided in DNeasy Blood and Tissue kit. The coding region of *spr cel8A* gene (GenBank accession number KX023906) (approx 1.1 kb) was amplified by PCR in a programmable thermal controller T100™ Thermocycler (BIO-RAD), using the HotStar Hifidelity, and the following pair of primers: forward, FCQE: 5′ATAGGATCC TGC GAG TGG CCG GCC TGG CAA C 3′, and reverse, RCQE: 5′ GCGAAGCTT TTC GGA AGT TAC GCA TTG GCC G 3′ with restriction sites *BamH*I and *Hin*dIII (underlined), respectively.

The reaction condition consisted of an initial 5 min step at 95 °C followed by 35 cycles of 15-s at 94 °C, 60-s at 82 °C and 90-s at 72 °C, with a final extension for 10-min at 72 °C. The amplicon of ≈1.1 kb was visualized on 0.8 % agarose gels stained with 10 μg/mL EtdBr and compared with a 1 kb base pair (bp) DNA ladder. The amplicon was purified with the QIAquick^®^ Gel extraction kit, cloned into pJET1.2/blunt vector, and transformed into chemically competent *E. coli* DH5α cells, generating the pJET1.2/*spr cel8A* construct.

### Construction of *spr cel8A* in the expression vector

The pJET1.2/*spr cel8A* construct was subjected to double digestion with *BamH*I and *Hin*dIII at 37 °C for 4 h, and the *spr cel8A* gene was directionally subcloned into the *BamH*I and *Hin*dIII restriction sites of the pQE30 expression vector. Recombinant plasmids (pQE30/*spr cel8A*) were propagated in *E. coli* JM109 and purified for DNA sequencing analysis. The pQE30/*spr cel8A* construct was transferred to *E. coli* M15 [pREP4] cell for protein expression assays.

### Expression of *Spr* Cel8A cellulase *in E. coli*

The recombinant cellulase was expressed from *E. coli* M15 [pREP4] cells harboring the pQE30/*spr cel8A* construct. Overnight *E. coli* cultures, grown in LB medium, were diluted 100-fold in LB medium and shake-incubated at 37 °C, 180 rpm, to a OD_600nm_ = 0.6 cell density. IPTG was added to reach a 1 mM final concentration and cultures were further incubated at 28 °C, 180 rpm, overnight. Subsequently, cell cultures were harvested by centrifugation (4000×*g* at 4 °C for 20 min).

### Preparation of cellulase crude extract

Cells contained in 1 mL of culture broth were harvested by centrifugation (4000×*g* at 4 °C for 20 min) and resuspended in Laemmli sample buffer, prior to being analyzed for total protein extract by 10 % SDS–PAGE following the Laemmli method (Laemmli 1970). Soluble protein was prepared from the bacterial pellet, of the remaining culture broth, resuspended in lysis buffer (100 mM NaCl, 2 mM EDTA and 50 mM Tris–HCl, pH 7.5) at 20:1 (w/v) ratio. Cells were lysed with lysozyme from chicken egg white (10 mg mL^−1^) at 4 °C for 30 min. Cell debris was removed by centrifugation (10,000×*g* at 4 °C for 15 min) and the supernatant, which was used as a source of crude cellulase was analyzed in order to reveal cellulase activity, as well as its protein profile, using 10 % SDS–PAGE.

### Protein electrophoresis

Protein analyses were carried out by 10 % SDS-PAGE using a Mini PROTEAN II system. Proteins in the gel were visualized by Coomassie Brilliant blue R-250. Protein molecular weight (MW) was estimated with reference to broad range molecular weight protein standards. Gels were recorded and analyzed using a gel documentation system (DigiDoc-It Im aging System, UVP). Protein concentration was determined as described by Lowry et al. (1951), using BSA as standard.

### Enzyme assay

The cellulolytic activity of *Spr* Cel8A on CMC was determined from the amount of reducing sugars released during incubation at 40 °C for 30 min. Reducing sugars were quantified using the dinitrosalicylic acid method, with glucose as standard (Miller 1959). A 20 μL of enzyme preparation was added to 1 mL of 50 mM citrate phosphate buffer, pH 7.0, containing 0.5 % (w/v) CMC as the substrate. One unit (U) of enzymatic activity was defined as the amount of enzyme required to produce 1 μmol of glucose per minute under assay conditions. All tests were carried out in triplicate and average values were recorded.

### Purification of a six His-tag recombinant enzyme

*Spr* Cel8A was purified by affinity chromatography from the soluble fraction of bacterial lysate (*E. coli* M15 [pREP4]/pQE30/*spr cel8A*), corresponding to cellulose crude extract.

Crude extract was loaded onto a Ni–NTA resin with a packed volume of 3.37 mL, previously equilibrated with buffer A (400 mM NaCl, 20 mM imidazole, 50 mM sodium phosphate buffer, pH 8.0). The C-terminal His_6_-tagged protein was subsequently eluted by linear gradient of 30–300 mM imidazole, using an Econo gradient pump system, at a constant flow rate of 1 mL min^−1^ and 1.0 mL fractions were collected. Fractions with cellulolytic activity were pooled, dialyzed against 50 mM citrate phosphate, pH 7.0, and analyzed by 10 % SDS-PAGE. The purified *Spr* Cel8A cellulase was stored at 4 °C for further study.

### Effect of pH on enzyme activity and stability

The effect of pH on the enzymatic activity of *Spr* Cel8A was evaluated at pH values, ranging from 3 to 10.5, in CMC (0.5 % w/v) substrate prepared in 50 mM of different buffers: citrate phosphate (pH 3–7), phosphates (pH 6–8) and glycin–NaOH (8.5–10.5), and incubated at 40 °C for 30 min. In order to evaluate the effect of pH on the stability of *Spr* Cel8A, samples of purified enzyme were incubated in a variety of buffers as mentioned previously, at pH values, ranging from 3.0 to 10.5, at 28 °C for 1 h. Subsequently, the remaining cellulolytic activity was measured under standard conditions and compared to untreated enzyme activity.

### Effect of temperature on enzyme activity and stability

The effect of temperature on enzymatic activity of purified *Spr* Cel8A was determined by conducting the assay at different temperatures ranging from 15 to 55 °C, in 0.5 % (w/v) CMC, dissolved in 50 mM citrate phosphate buffer, at a pH of 7.0, for 30 min. In order to evaluate enzyme thermostability, the purified *Spr* Cel8A was incubated at different temperatures (30, 40 and 50 °C) in 50 mM citrate phosphate buffer, pH 7.0. For determining the half-life of the enzyme, aliquots of the sample were withdrawn at different time intervals and residual enzymatic activity was measured under standard conditions and compared with the untreated enzyme activity.

### Substrate specificity and kinetic parameters

In order to evaluate substrate specificity of *Spr* Cel8A, cellulolytic activity was determined under optimal assay conditions using 0.5 % (w/v): birchwood xylan, oat spelt xylan, beechwood xylan, CMC, Avicel, Solka floc. For determining kinetic parameters *K*_*m*_ and *V*_*max*_, the initial reaction rates for *Spr* Cel8A were studied under optimal conditions for enzyme activity using CMC as substrate, at a concentration ranging from 0.2 to 1.2 mg. The *K*_*m*_ and *V*_*max*_ values were obtained by means of the nonlinear least squares regression method using the Michaelis and Menten kinetics (http://biomodel.uah.es/metab/enzimas/MM-regresion.htm).

### Effect of ions, EDTA and 2-mercaptoethanol (2-ME) on enzyme activity

In order to study the effect of metal ions and other compounds on the enzymatic activity of *Spr* Cel8A, samples of the purified enzyme were incubated in 0.5 % CMC prepared in 50 mM citrate phosphate buffer, pH 7.0 containing: Ca^2+^, Co^2+^, Mn^2+^, Ni^2+^, Mg^2+^, Fe^2+^, Cu^2+^, Na^1+^, Zn^2+^, Li^1+^, Hg^2+^; EDTA or 2-ME, at a final concentration of 1 and 5 mM each. Reaction mixtures were incubated with the different compounds at 28 °C for 60 min. Cellulase activities were assayed under standard conditions, and compared to a control without additions.

### Zymogram

Zymogram analysis was carried out as previously described (Schwarz et al. 1987), with some modifications as follows. Briefly, protein samples were separated in a 10 % polyacrylamide gel co-polymerized with 2 % CMC, under denaturing conditions. Protein samples were resuspended in SDS sample buffer without 2-β-mercaptoethanol, and the samples were boiled on waterbath for 5 min. After electrophoresis, the gels were incubated in 50 mM citrate–phosphate buffer, pH 7.0 at 40 °C for 30 min. Cellulase activity was visualized by staining the gel with Congo red (1 mg/mL) for 15 min, while being gently agitated, and then destained in 1 M NaCl for 10 min.

### Analysis of the hydrolysis products of recombinant cellulase using thin-layer chromatography

The degradation pattern and products of recombinant cellulase were analyzed using Thin Layer Chromatography (TLC). Briefly, 50 μL of purified *Spr* Cel8A cellulase was added to 50 μL of 1 % CMC in 50 mM citrate–phosphate buffer, pH 7.0. The reaction mix was incubated at 40 °C; and aliquot samples were taken for different time spans of between 0 and 144 h. A total of 3 μL of each sample and 2 μL of the cellulose-oligomer standard (glucose, cellobiose or cellotetraose) was spotted on silica gel. The hydrolysis products were separated in a solvent system consisting of butanol/ethanol/water (5:3:2 v/v/v). The plate was then sprayed with sulfuric acid (15 % v/v) and heated in a dry oven at 80 °C for 40 min to visualize the cellulose-oligomers.

### 3D model of *Spr* Cel8A from *S. proteamaculans*

The 3D structure model of endoglucanase was generated by submitting the amino acid sequence of *Spr* Cel8A from *S. proteamaculans* to the iterative threading assembly refinement (I-TASSER) server (http://zhanglab.ccmb.med.umich.edu/I-TASSER/).

## Results

### Analysis of the *spr cel8A* gene

**Fig. 1:**
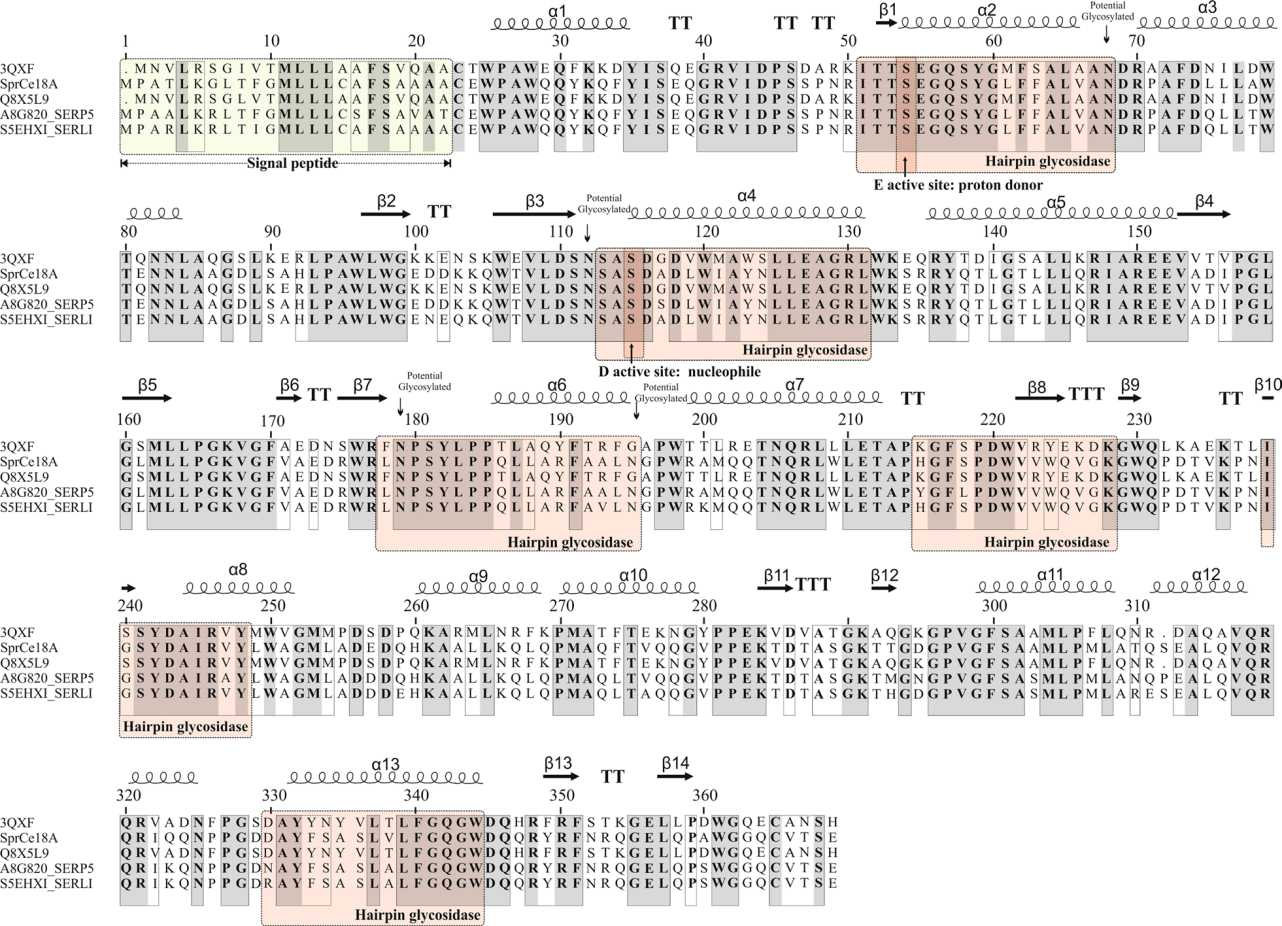
Multiple sequence alignment and secondary structure element assignment. The alignment included endoglucanases from *Serratia proteamaculans* strain 568 (A8G820, A8G820_SERP5), *S*. *liquefaciens* (S5EHX1, S5EHX1_SERLI) and *E. coli* O157 (Q8X5L9, GUN_ECO57). 3D crystal structure of the cellulase BcsZ from *Escherichia coli* strain K12 (PDB code 3QXF). Assignment of six hairpin glycosidase and active sites were manually determined for comparison with cellulase 3QXF from *E. coli* strain K12. The α helices are marked as α or β, based on the automatic assignment in conformity with the template of 3QXF protein structure in the ESPript program (Robert and Gouet 2014)

In order to putatively identify the predicted proteins involved in cellulose degradation, the genome of *S. proteamaculans* (strain DADMA8) was sequenced. Annotation and similarity analysis of the genome sequence allowed the identification of only one gene (*spr cel8A*), encoding for an endoglucanase precursor (EC 3.2.1.4). The *spr cel8A* gene from *S. proteamaculans* has an open reading frame (ORF) of 1113 bp, encoding for a predicted protein of 371 amino acid residues. The molecular mass and theoretical pI of the predicted *Spr* Cel8A were calculated to be 41.2 kDa and 5.37, respectively, with a signal peptide of 23 amino acids at the N-terminal end and four potential N-glycosylation sites (Fig. 1). Similarity analysis of the amino acid sequence for the predicted protein *Spr* Cel8A revealed that this enzyme is 93–94 % identical to endoglucanases belonging to the glycosyl hydrolase family 8 (GH8) from *S. proteamaculans* strain 568 (A8G820, A8G820_SERP5), *S. liquefaciens* (S5EHX1, S5EHX1_SERLI), *S. liquefaciens* (A0A109Z435, A0A109Z435_SERLI), and *S. grimesii* (A0A084YRC6, A0A084YRC6_9ENTR); and 59–61 % identical to GH8 endoglucanases from *E. coli* O157 (Q8X5L9, GUN_ECO57), *E. coli* strain K12 (P37651, GUN_ECOLI), *S. typhimurium* strain L72/SGSC1412/ATCC700720 (Q8ZLB7, GUN_SALTY) and *S. typhi* (Q8Z289, GUN_SALTI).

Alignment and comparison of the *Spr* Cel8A amino acid sequence deduced from *S. proteamaculans* with that of endoglucanase BcsZ sequence from *E. coli* strain K12 (P37651, GUN_ECOLI), that has been characterized and crystallized (PDB 3QXF), made possible the identification of 13-α helices, 14-β sheets, six-hairpin glycosidase-like (positions 52–65, 114–132, 179–196, 215–226, 240–249 and 331–354 aa), and a second hairpin (position 114–132), which corresponds to a conserved region in GH8. Amino acid residues E55 and D116 were identified as the proton donor and nucleophile respectively, because of their similarity (Fig. 1).

### Expression and purification of a six His-tag recombinant enzyme

**Fig. 2:**
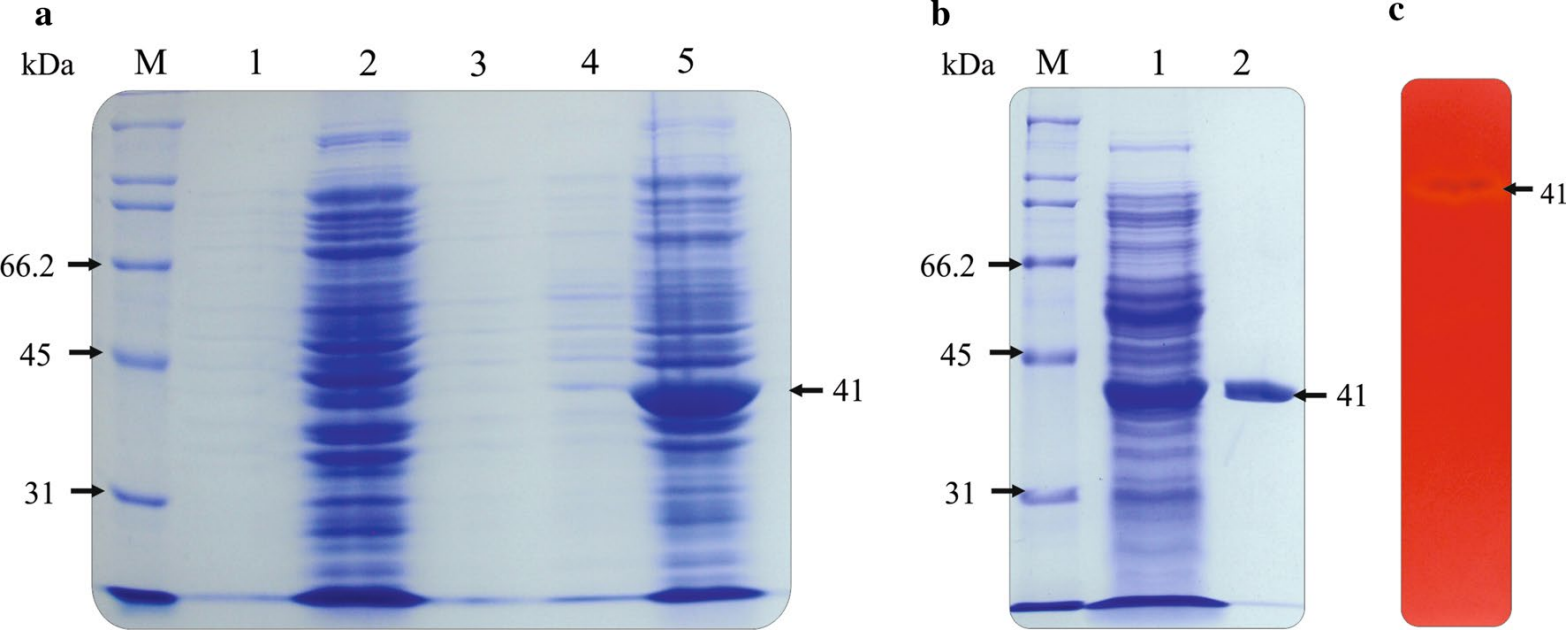
Protein and zymogram analysis of *Spr* Cel8A in 10 % SDS-PAGE. **a** Analysis of total protein extracts of transformed *E. coli* M15 [pREP4] /pQE30/spr cel8A, M: MW protein standards; *line 1 and 2* non-induced soluble fraction medium and bacterial cell harvested respectively; *line 3* induced soluble fraction medium; *line 4 and 5* induced lysed bacterial cells soluble fraction. **b** Purification of recombinant Spr Cel8A; *line 1* total protein extracts of transformed *E. coli* M15 [pREP4] /pQE30/Spr Cel8A and *line 2* Spr Cel8A purified. **c** Zimogram analysis of Spr Cel8A in 10 % SDS-PAGE with 2 % CMC

The coding region of the *spr cel8A* gene was expressed from *E. coli* M15 [pREP-4] cells, harboring the pQE30/*spr cel8A* construct, for functional analysis of the recombinant protein. The profile of total protein in the soluble fraction of the bacterial lysate revealed the overexpression of polypeptide with an estimated MW of 41 kDa, that corresponds to the expected MW for *Spr* Cel8A cellulase. In comparison to non-induced cells, the heterologous protein was expressed at high levels by cells, after induction with 1 mM IPTG (Fig. 2a, line 5). The recombinant protein was purified from the soluble fraction of the bacterial crude extract and purified by affinity chromatography on Ni–NTA resin. A summary of the purification steps for *Spr* Cel8A is presented in Table 1. SDS–PAGE analysis of extracts from the bacteria harboring the construct pQE30/*spr cel8A* revealed a single protein band of approximately 41 kDa, *Spr* Cel8A (Fig. 2b). The MW of *Spr* Cel8A conforms with the theoretical MW of the mature protein (39,008 Da), without the signal peptide. The zymogram analyses of purified *Spr* Cel8A, performed under denaturing conditions, showed a single band that confirmed the cellulolytic activity and monomeric nature of the enzyme (Fig. 2c).

**Table 1:** Purification of cellulase *Spr* Cel8A of *Serratia proteamaculans*

Step	Protein (mg)	Total activity (U)	Specific-activity (U/mg)	Purification fold	Recovery yield (%)
Soluble fraction of cell lysis	43.5	8.0	0.18	1	100
Ni–NTA affinity chromatography	1	0.85	0.85	4.72	10.58

### Biochemical characterization of *Spr* Cel8A protein

The purified *Spr* Cel8A cellulase from *S. proteamaculans* expressed in *E. coli* was biochemically characterized to provide insight concerning its biochemical properties and *K*_*m*_ and *V*_*max*_ kinetic parameters.

### Effect of pH on enzyme activity and stability

**Fig. 3:**
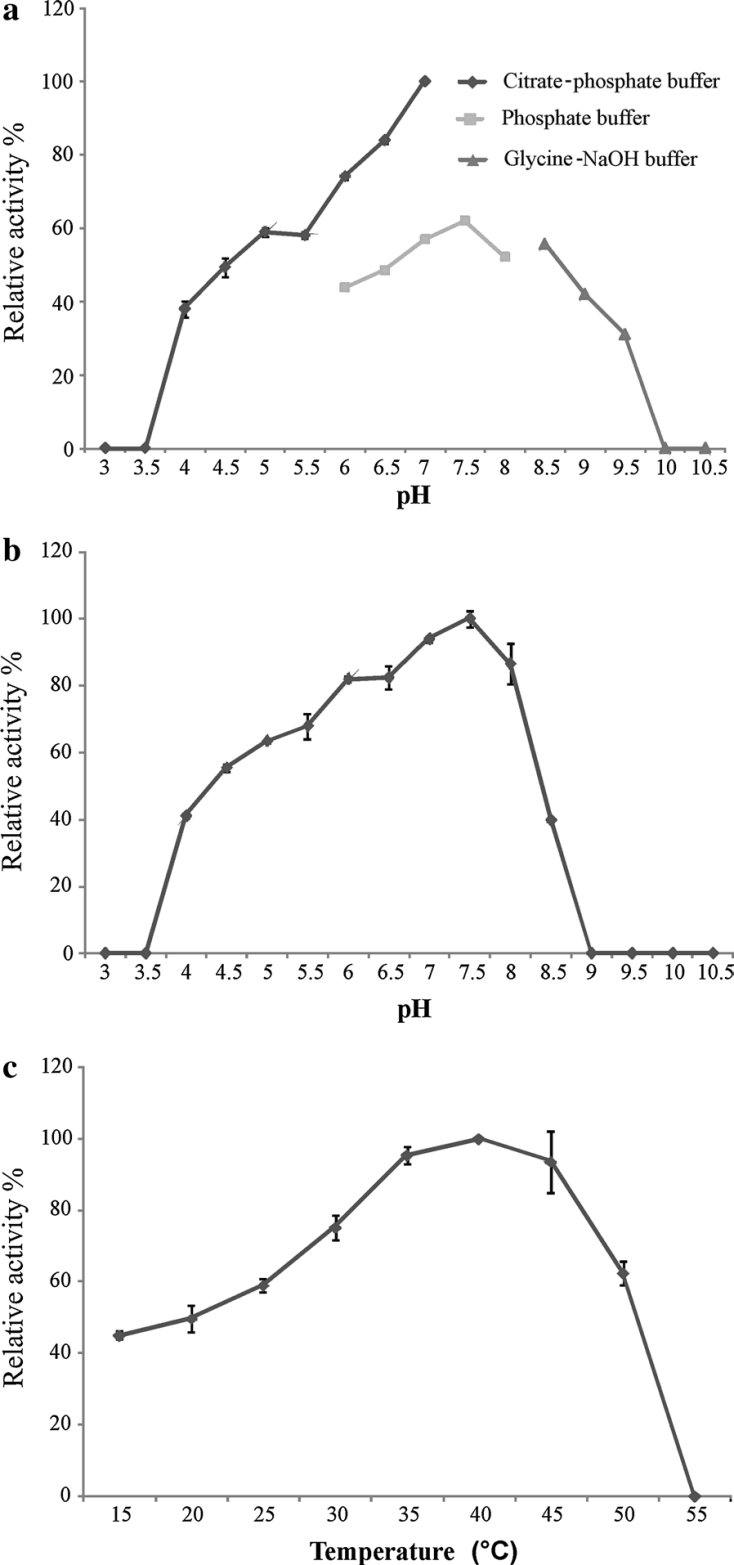
Effect of pH and temperature on *Spr* Cel8A activity and stability. **a** pH effect with different buffers; citrate phosphate (pH 3–7), phosphates (pH 6-8) and glycine-NaOH (8.5–10.5) and incubated at 40 ºC for 30 min. **b** pH stability, protein was incubated in different buffer, mentioned above, at different pH values, ranging from 3.0 to 10.5, at 28 ºC for 1 h. **c** Temperature effect, the enzyme was incubated with 0.5 % (w/v) CMC dissolved in 50 mM citrate phosphate buffer, pH 7.0, for 30 min at different temperatures (15–55 ºC). All tests were performed in triplicate and error bars indicate standard deviations

*Spr* Cel8A cellulase displayed optimal activity at pH 7 with a preference for citrate–phosphate buffer; however the enzyme exhibited 40–80 % of its maximal activity at different pH values ranging from 4 to 6.5, and 40 to 60 % at a pH of 7.5–9.5 (Fig. 3a). pH stability assays indicated that *Spr* Cel8A was highly stable at a pH range between 4 and 8.5, retaining 40–90 % of its original activity after 1 h of incubation at 28 °C (Fig. 3b).

### Effect of temperature on enzyme activity and stability

*Spr* Cel8A showed optimal activity at 40 °C (Fig. 3c); however, the enzyme manifested 40–90 % of its original activity at a wide range of temperatures, between 15 and 50 °C (Fig. 3c). The thermal stability of *Spr* Cel8A was studied at temperature values of 30, 40 and 50 °C at its optimal pH. The enzyme showed a half-life of 8 and 16 days at 40 and 30 °C, respectively (Table 2).

**Table 2:** Thermal stability of *Spr* Cel8A of *Serratia proteamaculans*

Temperature (°C)	Half life (t_1/2_)
30	16 days
40	8 days
50	25 min

### Substrate specificity and kinetic parameters

**Fig. 4:**
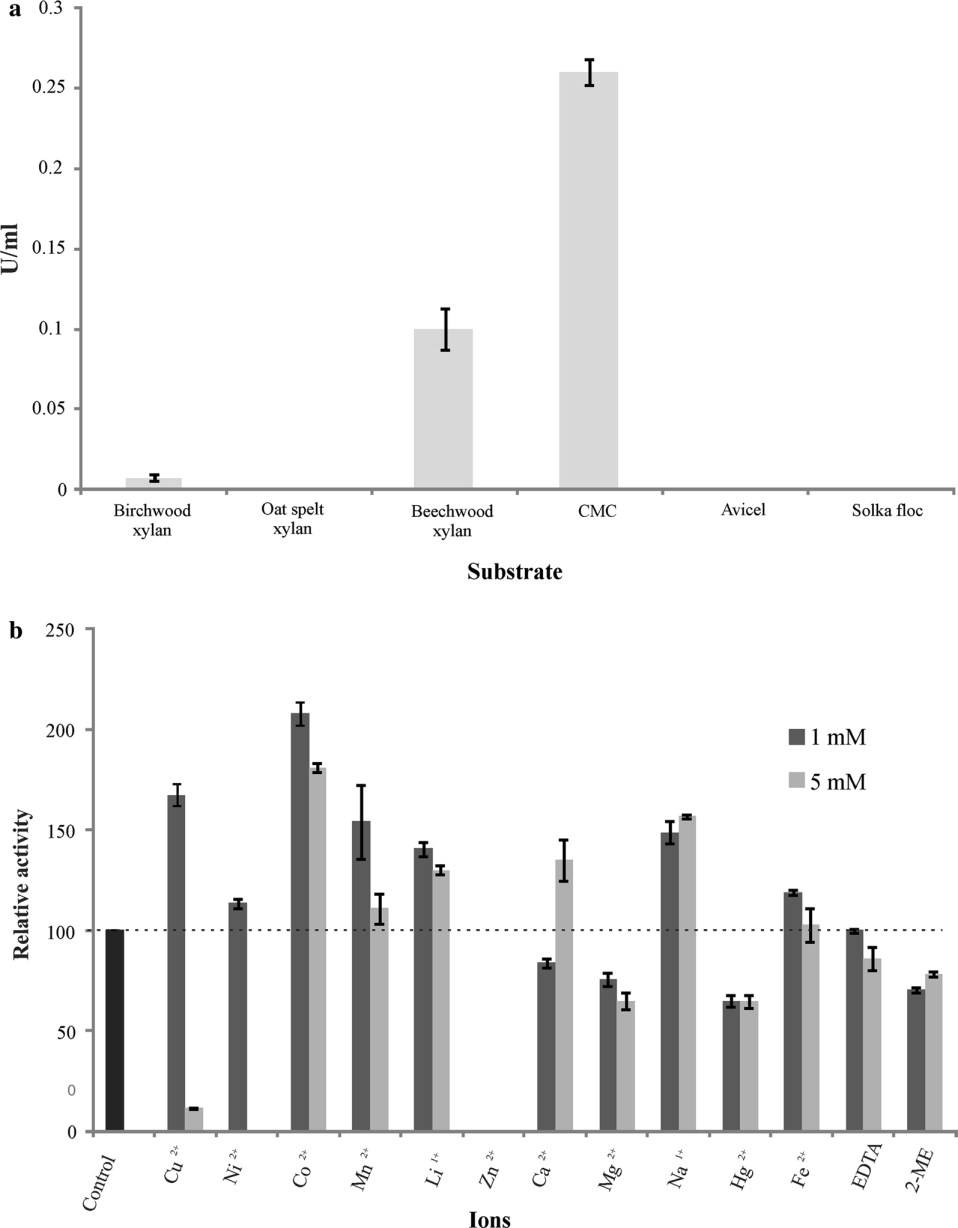
Substrate affinity of *Spr* Cel8A and effect of metal ions and other agents on enzyme activity. (**a**) The activity of *Spr* Cel8A was assayed using Avicel, CMC, Solka Floc, beechwood xylan, birchwood xylan, and oat spelt xylan as the substrate, at a final concentration of 0.5 % each in 50 mM citrate phosphate buffer, pH 7.0 at 40 ºC. (**b**) Effect of metal ions, EDTA and 2-ME on *Spr* Cel8A activity at two different concentrations 1 and 5 mM. Enzyme was incubated in 0.5 % (w/v) CMC in 50 mM citrate sodium phosphate buffer, pH 7.0 containing (1 and 5 mM each): Ca^2+^, Co^2+^, Mn^2+^, Ni^2+^, Mg^2+^, Fe^2+^, Cu^2+^, Na^1+^, Zn^2+^, Li^1+^, and Hg^2+^; EDTA and 2-ME at 28 ºC for 60 min. All tests were performed in triplicate and *error bars* indicate standard deviations

In order to evaluate the substrate specificity of *Spr* Cel8A, the activity of the enzyme was assayed using Avicel, CMC, Solka floc, beechwood xylan, birchwood xylan, and oat spelt xylan as the substrate, at a final concentration of 0.5 % each. *Spr* Cel8A showed higher affinity towards CMC, beechwood xylan and birchwood xylan, while no xylanase activity was detected in the presence of Avicel, Solka floc and oat spelt xylan (Fig. 4a). The kinetic parameters of *Spr* Cel8A were assayed under optimal pH and temperature conditions with CMC as the substrate, under different concentrations ranging from 0.2 to 1.2 mg/mL. The *K*_*m*_, *V*_*max*_ and *k*_*cat*_ (*V*_*max*_/[E]) were 6.87 mg/mL, 3.5 μmol/min/mg and 0.089 μmol/min, respectively.

### Effect of ions, EDTA and 2-mercaptoethanol on enzyme activity

The effect of metal ions, EDTA, and 2-ME, at 1 and 5 mM each, on the activity of *Spr* Cel8A was evaluated (Fig. 4b). *Spr* Cel8A activity was enhanced by 108 and 81 % with the ion Co^2+^ at 1 and 5 mM, respectively; whereas in the presence of the ion Cu^2+^ (1 mM), the activity of the enzyme increased by 67 %. The metal ions Mn^2+^ (1 mM), Li^1+^ (1 and 5 mM), Ca^2+^ (5 mM), and Na^1+^ (1 and 5 mM) increased the activity of *Spr* Cel8A by between 30 and 67 %. The activity of *Spr* Cel8A was almost completely inhibited in the presence of Zn^2+^ (1 and 5 mM), Ni^2+^ (5 mM) and Cu^2+^ (5 mM) (Fig. 4b); however, the enzyme displayed 64–78 % of its original activity in the presence of Mg^2+^, Hg^2+^ and 2-ME, at 1 and 5 mM each. In contrast, the metal ions Ni^2+^ (1 mM), Mn^2+^ (5 mM), Ca^2+^ (1 mM) and Fe^2+^ (1 and 5 mM), and the quelant agent EDTA (1 and 5 mM) had little or no effect on the activity of *Spr* Cel8A (Fig. 4b).

### Analysis of hydrolysis products by TLC

**Fig. 5:**
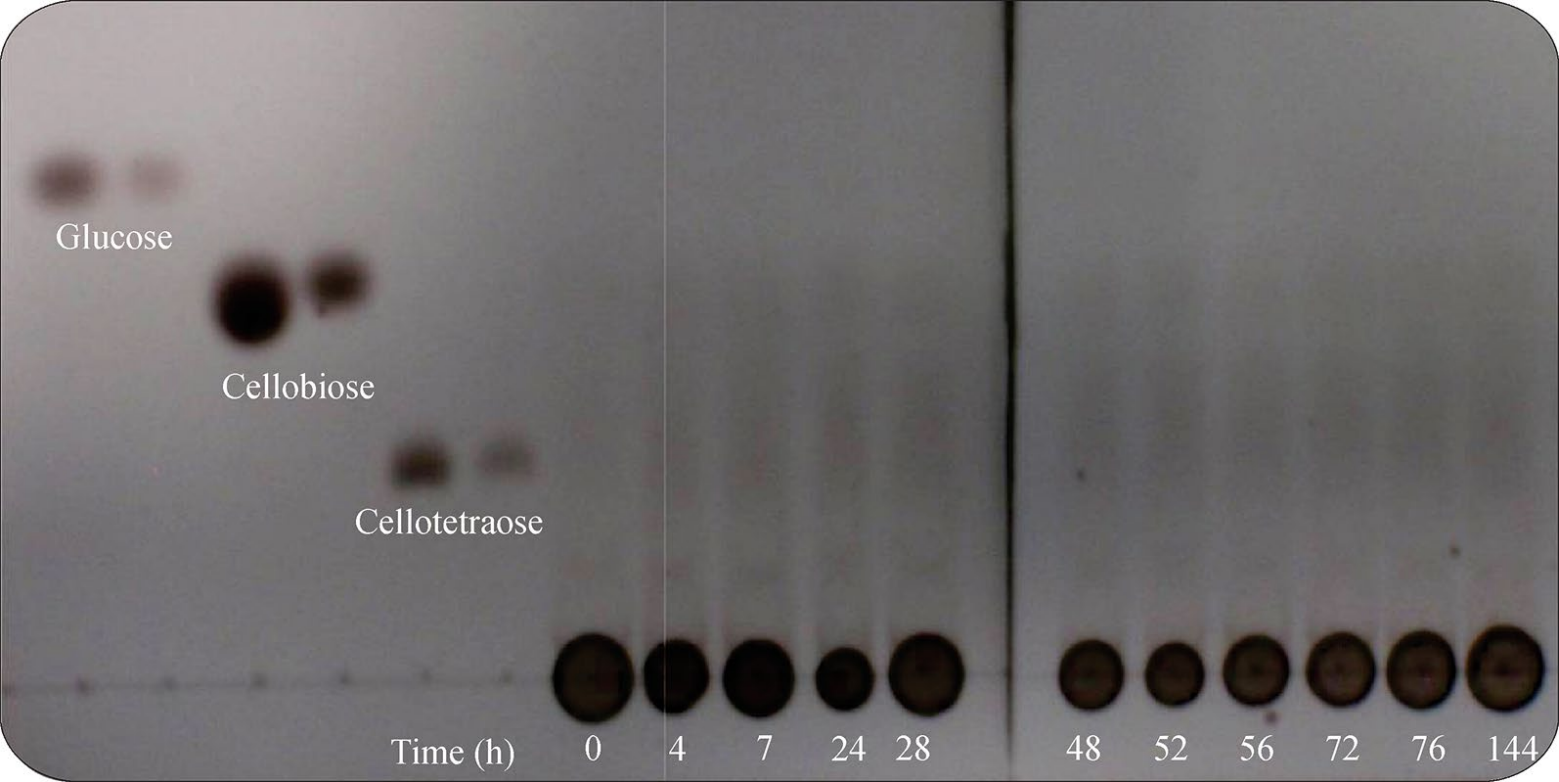
Thin layer chromatography of *Spr* Cel8A activity through cinetic time. Standards were presented at two concentrations

The hydrolysis products of CMC yielded by the action of *Spr* Cel8A for different incubation periods were analyzed by TLC. The hydrolysis products released were mainly cellobiose, unknown cello oligosaccharides and a small amount of glucose (Fig. 5).

### 3D model of *Spr* Cel8A

**Fig. 6:**
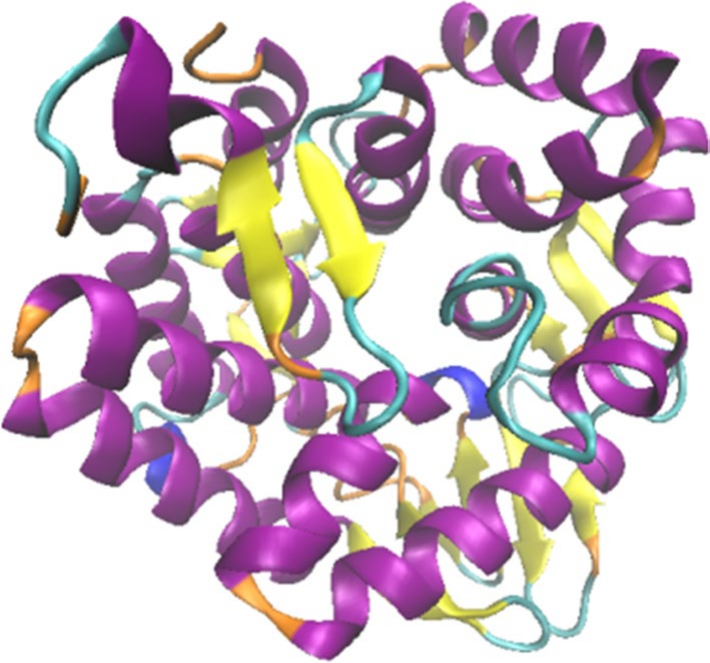
The predicted 3D model of *Spr* Cel8A from *S. proteamaculans* was obtained by the I-TASSER server. The α-helices are indicated in *purple*, 3_10 helix in *blue*, pi helix in *red*, β-sheets in *yellow*, turns in *cyan* and coils in *orange*

Initiating with the *Spr* Cel8A amino acid sequence from *S. proteamaculans*, the I-TASSER server generated a 3D structural model of *Spr* Cel8A with a C-score value of −0.02 (Fig. 6). The 3D model is structurally closest to the endo-1,4-β-d-glucanase from *Pseudomonas putida* KT2440 (PDB ID: 4Q2B) with a template modeling score (TM-score) of 0.90 and an alignment coverage of 0.90. The 3D model for *Spr* Cel8A from *S. proteamaculans* is very rich in α-helices with 50.54 %, followed by random coil structures with 42.71 %, and only about 6.75 % β-sheet content.

## Discussion

The metabolic capacities and functional role of gut-associated bacteria from bark beetles have not yet received extensive study, as has been undertaken for other insects such as termites, longhorn beetles, aphids, butterflies and moths (Watanabe and Tokuda 2010; Engel and Moran 2013). The cellulolytic activity of gut-associated bacteria from many insects has been well characterized by both qualitative and quantitative methods (Slaytor 1992; Sun and Scharf 2010); whereas in the case of bark beetles it has been only semiquantitatively determined by CMC degradation (Morales-Jiménez et al. 2009, 2012; Hu et al. 2014; Delalibera et al. 2005). In addition, no research has quantitatively evaluated this activity, or characterized the genes and corresponding enzymes.

In this study, we have identified, purified, characterized, and quantitatively evaluated the activity of an endo-β-1,4-glucanase from *S. proteamaculans,* isolated from the gut of the bark beetle *D. adjunctus*. *Serratia* spp. have been recurrently isolated from the gut of the *Dendroctonus* species (Morales-Jiménez et al. 2009; Hu et al. 2014; Vasanthakumar et al. 2006; Xu et al. 2015), and their relative abundance and persistence in this habitat suggest that they are part of the microbiome core of these insects (Hernández-García, personal communication). Thus, it appears probable that the cellulolytic activity from *Serratia* sp. makes a quantitative substantial contribution to the nutrition of these insects.

The complete genome analysis of *S. proteamaculans* indicates that it contains only a single gene (*spr cel8A*) involved in cellulose hydrolysis, which has only been observed in 38 % of bacterial genomes deposited in the carbohydrate-active enzyme database (CAZy). For this reason this species was selected for being non saprophytic bacteria that can hydrolyse and synthesize cellulose, as it has an operon which can biosynthesize this polymer, also present in the *S. proteamaculans* 568 strain (Medie et al. 2012).

The predicted product of the *spr cel8A* gene, *Spr* Cel8A, from *S. proteamaculans* identified in this study shows conserved residues characteristic of the GH8 family (Fig. 1), thus suggesting that *Spr* Cel8A can be classified as endoglucanase pertaining to the GH8 family. The 3D model of *Spr* Cel8A from *S. proteamaculans* (Fig. 6) predicts a 3D structure (α/α)_6_, closely related to the 3D structure models (α/α)_6_ of the GH8 endoglucanase family from *P. putida* KT2440 (UniProtKB accesion code: Q88JL2_PSEPK) and *E. coli* K-12 (Mazur and Zimmer 2011). Concurring with our results, it has been reported that endoglucanases belonging to the GH8 family present a 3-D structure (α/α)_6_ (Taylor and Vaisman 2010).

Twenty six endocellulases from the GH8 family have been characterized in terms of bacteria (Berlemont and Martiny 2013), but none of these have been associated with species from the *Serratia* genus. Therefore, this is the first report of a endo-1,4-β-glucanase from the GH8 family associated with a *Serratia* species. Similarly, based on molecular and functional characteristics, the endo-1,4-β-glucanase *Spr* Cel8A from *S. proteamaculans* should be classified within clan M of the GH8 family, as these are involved in hydrolysis of cellulose by means of a reaction mechanism for inversion (Henrissat 1991; Yennamalli et al. 2011).

Likewise, amino acid identity analyses show that *Spr* Cel8A endo-1,4-β-glucanase from *S. proteamaculans* is 94–95 % similar to other endoglucanases annotated from *Serratia* spp. (Fig. 1), which have not been biochemically characterized. The endo-1,4-β-glucanase *Spr* Cel8A from *S. proteamaculans* is 61 % identical to the endoglucanase from *E. coli* strain K12, whose 3-D structure has been determined by X-ray crystallography (Mazur and Zimmer 2011).

Recombinant endo-1,4-β-glucanase *Spr* Cel8A from *S. proteamaculans* displayed remarkable biochemical characteristics that may optimize its performance within the insect’s gut. These include, optimal activity recorded at pH 7.0, cellulolytic activity exhibited over a wide range of pH (4.5–9.5) (Fig. 3a), as well as its high stability within this potential hydrogen interval (Fig. 3b). This suggests that the enzyme may be functional within all gut regions of bark beetles. Our results coincide with that observed in other bacteria such as *Komagataeibacter xylinus* (Koo et al. 1998) and *Bacillus* sp. HSH-810 (Kim et al. 2005) isolated from other environments, whose endo-1,4-β-glucanases also manifest optimal activity at pH 7.0 and notable activity at a wide pH ranging from 4.5 to 10. However, these characteristics may apparently constitute an exception, because the rule is that bacterial endo-1,4-β-glucanases work at an optimal pH, but do not show activity at wide intervals of pH, as has been documented in *Sporocytophaga myxococcoides* (Goksoyr 1988), *Pseudomonas fluorescens* (Yamane et al. 1970); *Ruminiclostridium thermocellum* (Romaniec et al. 1992), *Aquifex aeolicus* (Kim et al. 2000), *Ruminococcus albus* (Ohara et al. 2000), *Cellulomonas biazotea* (Rajoka et al. 2004), *Clostridium cellulovorans* (Arai et al. 2006), *Bacillus mycoides* (Balasubramanian et al. 2012), and *Bacillus* sp. (Harshvardhan et al. 2013).

Endoglucanase *Spr* Cel8A showed pH stability over a wide range of pH from 4 to 8.5; similar results were found among endoglucanases from *Bacillus amyloliquefaciens* (Lee et al. 2008) and the archaeon from *Pyrococcus horikoshii* (Yang et al. 2012), with pH stability for a pH range of 4–9.0.

Another interesting biochemical characteristic is the optimal temperature of the β-1,4-endoglucanase *Spr* Cel8A from *S. proteamaculans*, with an optimal activity at 40 °C, which maintains 40–90 % of its maximal activity in the temperature range from 15 to 50 °C (Fig. 3c). The optimum temperature recorded for different bacterial endoglucanases shows broad temperature values, ranging from 25 °C in *Fibrobacter succinogenes* (Lyo and Forsberg 1996) to 100 °C in *Thermotoga maritima* (Cheng et al. 2012). Findings here indicate that the *Spr* Cel8A endoglucanase from *S. proteamaculans* is a mesophilic enzyme.

Thermostability assays indicated that *Spr* Cel8A displays low thermostability at 50 °C; however the enzyme exhibited half-lifes of 16 and 8 days at 30 and 40 °C, respectively. Findings here are comparable to that reported for the commercial cellulase from *Trichoderma reesei*, whose activity decreases by 15 % after 150 h in the temperature range from 30 to 50 °C (Balsan et al. 2012). Interestingly, the thermostability of *Spr* Cel8A at 30 and 40 °C is an attribute that should be emphasized because bark beetle insects spend much of their life cycle under the bark of pine trees feeding on phloem (Wood 1982), where the interior temperature can range between 15 and 40 °C, and their gut temperature varies from 12 to 33 °C (Wermelinger and Seifert 1998). This temperature range conforms to the temperature at which all insect life stages, including egg and adult growth, and development and performance of metabolic processes such as nutrition, take place.

*Spr* Cel8A was able to hydrolyze CMC and to a lesser extent, beechwood xylan (38 %) and birchwood xylan (2.9 %); however the enzyme was not active on Avicel, Solka floc and oat spelt xylan (Fig. 4a). The *K*_*M*_ value of *Spr* Cel8A was 6.87 mg/mL, which is comparable to that reported for the halophilic cellulase CelB from *Bacillus* sp. BG-CS10, with a *K*_*M*_ value of 6.6 mg/mL, using CMC as substrate with no NaCl (Zhang et al. 2012). However, *K*_*M*_ values ranging from 0.01 to 6.6 mg/ml have been reported for endoglucanases, using CMC as the substrate (Zhang et al. 2012; Kupsky et al. 2014).

It is generally accepted that some metal ions and reagents significantly affect cellulase activities (Wang et al. 2012). Therefore, the effect of metal ions, EDTA and 2-mercaptoethanol on the activity of *Spr* Cel8A was evaluated. *Spr* CelA from *S. proteamaculans* was inhibited Zn^2+^ (1 and 5 mM) or almost completely inhibited by Cu^2+^ at 5 mM (Fig. 4b). The inhibitory effect of Cu^2+^ and Zn^2+^ on the activity of cellulases and xylanases is a common feature among these enzymes (Romaniec et al. 1992; Paradis et al. 1993). However, the activity of *Spr* Cel8A increased in the presence of Co^2+^, Mn^2+^, Li^1+^ and Na^1+^ (1 and 5 mM), and other metallic ions such as Cu^2+^, Ni^2+^ and Fe^2+^ at 1 mM, and Ca^2+^ 5 mM. This suggests that this endoglucanase manifests a conformational stability, because the enzyme retained its activity even in the absence of metallic ions (Welfle et al. 1995; Rubini et al. 2010). An increase of 139.5 % in the activity of an endo-1,4-β-glucanase from *B. subtilis* was observed in the presence of Co^2+^ (Au and Chan 1987); whereas *Spr* CelA from *S. proteamaculans* increased by 108 %. In *Clostridium thermocellum* it has also been documented that Ca^2+^ stabilizes the endoglucanase structural conformation because it has higher affinity for the substrate and great thermostability (Chauvaux et al. 1990). Notably, the enzyme displayed 64–78 % of its original activity in the presence of Mg^2+^, Hg^2+^, 2-ME and EDTA at 1 and 5 mM each (Fig. 4b). Enzyme inhibition by metallic cations usually suggests the presence of at least one sulfhydryl group in the active site, usually a cysteine amino acid (Rubini et al. 2010). This group oxidation by the cations destabilizes the conformational folding of the enzyme (Rouvinen et al. 1990) or leads to the formation of disulfide bonds at an irregular position of the protein (Ohmiya et al. 1995). Therefore, the capacity of *Spr* Cel8A from *S. proteamaculans* to remain active in the presence of several metal ions and chemical agents, including Hg^2+^, EDTA and 2-ME, is a fundamental characteristic because many ions are present in the phloem of trees.

Concerning the final hydrolysis products, *Spr* CelA from *S. proteamaculans* can be classified as an endo-β-1,4-glucanase that undergoes exo-β-1,4-glucanase activity due to its ability to release cello oligosaccharides, cellobiose and glucose from CMC. Other endo-β-1,4-glucanases that release cello oligosaccharides, cellobiose and glucose from CMC as the main hydrolysis products have been reported previously. For instance, the E4-90 endo-β-1,4-cellulase from *Thermomonospora fusca* releases mainly cellobiose, cellotriose, cellotetraose and glucose with CMC as the substrate, suggesting that this enzyme is involved in endo- and exo-activity (Irwin et al., 1998), and the endoglucanase Cel9A from *Thermobifida fusca* releases mainly cellobiose, with some minor products e.g., cellopentaose, cellotetraose, cellotriose with CMC as the substrate (Chir et al. 2011). In contrast, the endocellulase Cel5A from *Cellvibrio mixtus* releases mainly cellobiose and cellotriose with no evidence of glucose formation during the early stage (Voget et al. 2006).

In summary, we have successfully expressed the endoglucanase *Spr* Cel8A from *S. proteamaculans* in *E. coli*. This enzyme is neutral, mesophilic, displays high affinity to CMC polysaccharide and exhibits exo-activity. Interestingly, *Spr* Cel8A is highly active in a wide range of pH values (4–9.5) and temperatures (15–50 °C), and has higher thermostability than other cellulases previously reported, such as the commercial cellulose from *T. reesei.* Additionally*, Spr* Cel8A is stable in the presence of several metal ions, including Hg^2+^. This characteristics made this enzyme suitable for structure–function studies. Although many different bacteria endoglucanases have been identified and cloned, only a few are from the genus *Serratia*, and this is the first report of isolation, cloning and expression of an endo-β-1,4-glucanase from *S. proteamaculans,* pertaining to the gut-associated bacteria community in *Dendroctonus* bark beetles. The identification and characterization of this enzyme may improve understanding of the functional role of *S. proteamaculans* as symbiont of bark beetles.

## Abbreviations

SDS-PAGE: sodium dodecyl sulfate polyacrylamide gel electrophoresis
BSA: bovine serum albumin
PBS: phosphate-buffered-saline
TSA: tryptic soy agar
CMC: carboxymethyl cellulose
LB: Luria–Bertani
RAST: Rapid Annotation Subsystem Technology
bp: base pair
IPTG: Isopropyl-β-d-thiogalactopyranoside
MW: molecular weight
pI: isoelectric point
U: unit
EDTA: ethylenediaminetetraacetic acid
2-ME: 2-mercaptoethanol
TLC: Thin Layer Chromatography
GH8: glycosyl hydrolase family 8
CAZy: carbohydrate-active enzyme database
